# Inhibition of aromatase activity and expression in MCF-7 cells by the chemopreventive retinoid *N* -(4-hydroxy-phenyl)-retinamide

**DOI:** 10.1054/bjoc.2000.1269

**Published:** 2000-07-03

**Authors:** H P Ciolino, T T Y Wang, N Sathyamoorthy

**Affiliations:** Cellular Defense and Carcinogenesis Section, Basic Research Laboratory, Division of Basic Sciences, National Cancer Institute-Frederick Cancer Research and Development Center, Frederick, Maryland, 21701–1201, USA; Phytonutrients Laboratory, US Department of Agriculture, Beltsville Human Nutrition Research Center, Beltsville, MD, 20705, USA; Tumour Biology and Metastasis Branch, National Cancer Institute, Rockvile, MD, 20892, USA

**Keywords:** aromatase, 4-HPR, MCF-7, cAMP

## Abstract

The effect of the chemopreventive synthetic retinoid *N* -(4-hydroxyphenyl)-retinamide (4-HPR) on aromatase activity and expression was examined. 4-HPR caused a dose-dependent inhibition of aromatase activity in microsomes isolated from JEG-3 human placental carcinoma cells. The kinetics of inhibition were analysed by double-reciprocal plot. The *K* m of the substrate increased and the *V* max of the reaction decreased in the presence of 4-HPR, indicating that enzyme inhibition involved both competition for the substrate-binding site and non-competitive mechanisms. To determine whether 4-HPR would also inhibit aromatase activity in intact cells, MCF-7 human breast cancer cells were incubated with or without cAMP in the presence of 4-HPR. 4-HPR inhibited both basal and cAMP-induced aromatase activity in intact MCF-7 cells. The induction of aromatase mRNA expression in MCF-7 cells by cAMP was inhibited in cells treated with 4-HPR. These results indicate that 4-HPR inhibits both the enzymatic activity and expression of aromatase. These activities may play an important role in the known chemopreventive effect of 4-HPR towards breast cancer. © 2000 Cancer Research Campaign


*British Journal of Cancer* (2000) **83**(3), 333–337
© 2000 Cancer Research Campaign
doi: 10.1054/ bjoc.2000.1269, available online at http://www.idealibrary.com on IDEAL®

### Inhibition of aromatase activity and expression in MCF-7 cells by the chemopreventive retinoid *N*-(4-hydroxy-phenyl)-retinamide

**HP Ciolino[1], TTY Wang[2] and N Sathyamoorthy[3]**

[1]Cellular Defense and Carcinogenesis Section, Basic Research Laboratory, Division of Basic Sciences, National Cancer Institute-Frederick Cancer Research and Development Center, Frederick, Maryland 21701–1201, USA; [2]Phytonutrients Laboratory, Beltsville Human Nutrition Research Center, US Department of Agriculture, Beltsville, MD 20705, USA; [3]Tumour Biology and Metastasis Branch, National Cancer Institute, Rockvile, MD 20892, USA

**Summary** The effect of the chemopreventive synthetic retinoid *N*-(4-hydroxyphenyl)-retinamide (4-HPR) on aromatase activity and expression was examined. 4-HPR caused a dose-dependent inhibition of aromatase activity in microsomes isolated from JEG-3 human placental carcinoma cells. The kinetics of inhibition were analysed by double-reciprocal plot. The *K*m of the substrate increased and the *V*max of the reaction decreased in the presence of 4-HPR, indicating that enzyme inhibition involved both competition for the substrate-binding site and non-competitive mechanisms. To determine whether 4-HPR would also inhibit aromatase activity in intact cells, MCF-7 human breast cancer cells were incubated with or without cAMP in the presence of 4-HPR. 4-HPR inhibited both basal and cAMP-induced aromatase activity in intact MCF-7 cells. The induction of aromatase mRNA expression in MCF-7 cells by cAMP was inhibited in cells treated with 4-HPR. These results indicate that 4-HPR inhibits both the enzymatic activity and expression of aromatase. These activities may play an important role in the known chemopreventive effect of 4-HPR towards breast cancer. © 2000 Cancer Research Campaign

**Keywords**: aromatase; 4-HPR; MCF-7; cAMP

Aromatase is the product of the *CYP19* gene, a member of the P450 superfamily of genes. It catalyses the rate-limiting step in oestrogen biosynthesis, the conversion of $C_{19}$ androgenic steroids to the corresponding oestrogen, a reaction termed aromatization since it converts the $\Delta^4$-3-one A-ring of the androgen to the phenolic A-ring of oestrogen (Simpson et al, 1997). Several studies have demonstrated measurable levels of aromatase mRNA and enzyme activity in breast cancer tissues (Brodie et al, 1997; Santner et al, 1997). As a result of in situ oestrogen production by aromatase, breast tumour oestrogen concentrations remain high, even in post-menopausal women (Miller and Forrest, 1976). Aromatase activity in tumours or surrounding tissue may play a significant role in promoting tumour growth due to this local production of oestrogen (Brodie et al, 1997; Sasano and Harada, 1998; Blankenstein et al, 1999), which has been demonstrated to have potent mitogenic activity on breast cancer cells in vitro (Prall et al, 1998). Indeed, the majority of breast tumours are oestrogen-responsive (de Cupis and Favoni, 1997). Therefore, one promising approach to breast cancer therapy is to reduce or eliminate oestrogen production by aromatase. Several aromatase inhibitors have been synthesized and are in clinical trials (Brodie et al, 1999).

Retinoids, which include natural vitamin A (retinol), its metabolites and esters, and synthetic analogues, are among the most well-studied agents in chemoprevention. A number of retinoids have significant preventive activity in many in vivo experimental systems against breast, skin, bladder, lung, and oral carcinogenesis (Sankaranarayanan and Mathew, 1996). In clinical trials, several retinoids have achieved significant activity in the reversal of head and neck, skin and cervical premalignancy, and in the prevention of secondary tumours associated with breast, head and neck, skin and non-small cell lung cancers (Man, 1994; Lippman et al, 1995). In particular, a synthetic analogue of retinoic acid, *N*-(4-hydroxyphenyl)retinamide (4-HPR), also known as fenretinide, has been extensively studied, as it is less toxic at pharmacological doses in humans than natural retinoids. 4-HPR has been shown to have many biological activities in vitro and in vivo which may result in a decrease in the development and progression of breast cancer. 4-HPR has potent preventive effects in rodent mammary tumour models (Crist et al, 1997; Moon and Constantinou, 1997). Interestingly, 4-HPR has been found to accumulate preferentially in breast tissue (Formelli et al, 1993). Based on its low toxicity profile, it is currently being tested in a large breast cancer prevention trial (Costa et al, 1994). A definitive mechanism by which 4-HPR exerts its chemopreventive effect on mammary tumour growth has not been identified; indeed, in vitro experiments suggest that 4-HPR exerts multifaceted effects on several pathways important to carcinogenesis and tumour progression, including suppression of cell growth (Marth et al, 1985), induction of apoptosis (Wang and Phang, 1996), inhibition of telomerase activity (Bednarek et al, 1999) and inhibition of insulin-like growth factor-induced cell growth (Favoni et al, 1998).

Because of the importance of aromatase to the growth of breast tumours, in the present study we have examined the effect of 4-HPR on aromatase activity and expression. In order to study the direct effect of 4-HPR on aromatase activity, we have used the microsomal fraction isolated from JEG-3 human choriocarcinoma

*Received 31 August 1999*
*Revised 10 March 2000*
*Accepted 24 March 2000*

*Correspondence to:* HP Ciolino cells as a source of aromatase. To study the effect of 4-HPR on cellular aromatase activity and expression, we used the MCF-7 human breast cancer cells, which have been extensively used in aromatase studies (Yano et al, 1995; Zhou et al, 1996). We report, for the first time, that 4-HPR inhibits microsomal and cellular aromatase activity, and inhibits the expression of aromatase mRNA induced by cAMP.

### MATERIALS AND METHODS

### Materials

Unless otherwise noted, all reagents were from Sigma (St. Louis, MO, USA). 4-HPR was dissolved in dimethylsulphoxide (DMSO) at a concentration of 100 mM, aliquoted, and stored at –20°C. All culture media components and trypsin/EDTA were from BioFluids (Rockville, MD, USA).

### Cell culture

JEG-3 human choriocarcinoma cells and MCF-7 human breast carcinoma cells were purchased from the American Type Culture Collection (Rockville, MD, USA). JEG-3 cells were maintained in Eagle's minimum essential medium with non-essential amino acids, supplemented with 10% foetal bovine serum, and 2 mM glutamine. MCF-7 cells were grown in RPMI 1640 supplemented with 10% foetal bovine serum and 2 mM glutamine. Both cell lines were passed weekly using 0.05% trypsin, 0.02% EDTA.

### Microsomal preparation

JEG-3 cells were grown to confluence in 175 $cm^2$ culture flasks. The cells were trypsinized and pelleted by centrifugation, and the pellet resuspended with phosphate-buffered saline (PBS) and repelleted. The cell pellet was resuspended in 10 mM Tris-HCl, pH 7.5, containing 0.25 M sucrose and protease inhibitors (100 μg $ml^{-1}$ phenylmethysulphonyl fluoride, 300 μg $ml^{-1}$ EDTA, 0.5 μg $ml^{-1}$ leupeptin, 0.5 μg $ml^{-1}$ aprotinin and 0.7 μg $ml^{-1}$ Pepstatin A). The cells were sonicated for 30 s on ice with a Branson sonifer, setting 2. The sonicate was then subjected to centrifugation at 10 000 ***g***, 4°C, for 10 min to remove cellular debris. The supernatant was subjected to centrifugation at 500 000 ***g***, 4°C, for 15 min. The supernatant was removed and the microsomal pellet was resuspended in the above buffer. Protein was measured by the method of Bradford (1976) using bovine serum albumin as a standard. Microsomes were aliquoted and stored at –70°C.

### Measurement of microsomal aromatase activity

Microsomal aromatase activity was measured by the $^3H_2O$ release method as described by Choate and Resko (1996). This assay measures the amount of $^3H_2O$ formed during the conversion of androstenedione to estrone by aromatase. Briefly, 10 μg of JEG-3 microsomes were incubated with DMSO (control) or 4-HPR for 1 h at 37°C in 275 μl of PBS, pH 7.5, with 1 mM NADPH and 25 nM [1β-$^3$H(N)]-androst-4-ene-3,17-dione (NEN, Boston, MA, USA). The reaction was terminated by the addition of 75 μl of 50% trichloroacetic acid. This was subjected to centrifugation at 15 000 ***g***, 4°C, for 15 min. The supernatant (275 μl) was removed and 80 μl of 10% activated charcoal and 45 μl $H_2O$ was added. This was vortexed gently and incubated at room temperature for 30 min. It was then subjected to centrifugation at 15 000 ***g***, 4°C, for 15 min. Radioactivity released as $^3H_2O$ was determined by scintillation counting of 100 μl of the supernatant in Aquasol scintillation fluid (Beckman, Palo Alto, CA, USA).

To determine the mechanism of inhibition, microsomal aromatase activity was determined as described above in the presence of different concentrations of [1 β-$^3$H(N)]-androst-4-ene-3, 17-dione with or without 4-HPR and a double-reciprocal (Lineweaver–Burke) plot was generated.

### Measurement of cellular aromatase activity

The amount of aromatase activity in intact MCF-7 cells was measured as described in Shimodaria et al (1996). Confluent MCF-7 cells in six-well culture plates were incubated at 37°C for 24 h in 1 ml of growth medium containing 25 nM [1 β-$^3$H(N)]-androst-4-ene-3,17-dione with or without 1 mM dibutryl-cyclic AMP (cAMP) in the presence of DMSO (control) or 4-HPR. The medium was then removed and 250 μl of activated charcoal was added. This was incubated for 30 min at room temperature and subjected to centrifugation at 15 000 ***g***, 4°C, for 15 min. 625 μl was used for scintillation counting as described above.

### Measurement of aromatase mRNA

The amount of aromatase mRNA was determined by semi-quantitative reverse transcription-polymerase chain reaction (RT-PCR). Confluent MCF-7 cells in six-well culture plates were incubated at 37°C for 24 h in 1 ml of growth medium with or without 1 mM cAMP in the presence of DMSO (control) or 4-HPR. Following incubation, the cells were washed with PBS and total RNA was isolated using Trizol reagent (Life Technologies, Gaithersburg, MD, USA) as directed. cDNA was synthesized from 10 μg of total RNA using a RT-PCR kit from Stratagene (La Jolla, CA, USA) as instructed. PCR was performed using the aromatase primer sequences and method of Zhou et al (1990), in the presence of 1.5 μCi of [$^{32}$P]dATP (NEN). PCR was also run using primers for glucose-3-phosphate dehydrogenase (G-3-PDH; Clonetech, Palo Alto, CA, USA) as directed. The optimum cycle number that fell within the exponential range of response for aromatase (25 cycles) and G-3-PDH (19 cycles) was used. Following PCR, 5 μl of high-density buffer (Novex, San Diego, CA, USA) was added to the samples, and they were subjected to electrophoresis on a 10% tris-borate EDTA gel (Novex) in 1× tris-borate EDTA buffer (Novex) for 1.5 h at 125 V. The gel was then dried and visualized by phosphoimaging using a Bio-Rad GS-363 phosphoimager (Hercules, CA, USA).

### Statistical analysis

Statistical analysis was performed using one-way analysis of variance (ANOVA), followed by the Fischer test. A $P$-value of $<0.05$ was considered to be significant.

### RESULTS

### Effect of 4-HPR on microsomal aromatase activity

Microsomes isolated from JEG-3 human placental cancer cells were used to determine the direct effect of 4-HPR on aromatase activity. Incubation of 10 μg microsomes with 25 nM of the

*© 2000 Cancer Research Campaign*

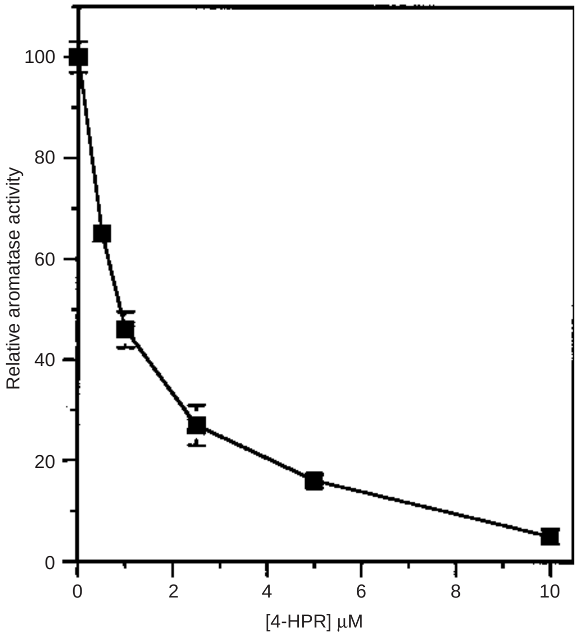

**Figure 1** Effect of 4-HPR on microsomal aromatase activity. 10 µg of microsomes isolated from JEG-3 cells were incubated with 25 nM [1β-$^3$H(N)]-androst-4-ene-3,17-dione and 1 mM NADPH for 1 h and the amount of $^3H_2O$ released was measured as described. Each point represents the mean of four determinations ± standard error (SE). There was a statistically significant decrease in aromatase activity at all 4-HPR concentrations tested ($P < 0.05$)

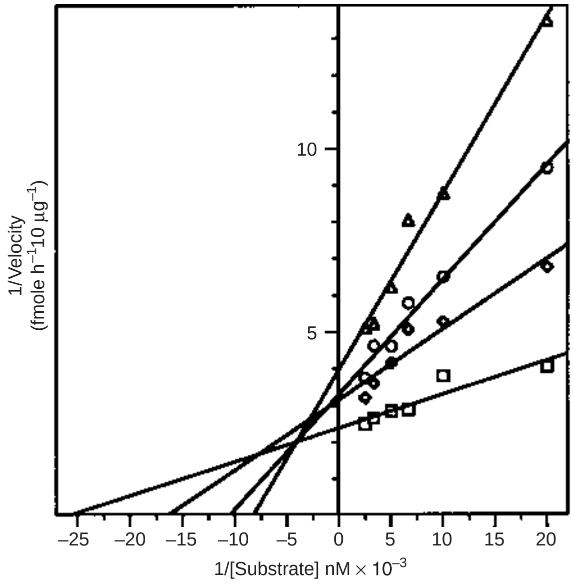

**Figure 2** Effect of 4-HPR on microsomal aromatase activity in the presence of different substrate concentrations. 10 µg of JEG-3 microsomes were incubated with NADPH and the indicated concentrations of [1β-$^3$H(N)]-androst-4-ene-3,17-dione in the presence of 0 (squares), 1 (diamonds), 2.5 (circles), or 5 (triangles) µM 4-HPR. Aromatase activity was measured as described and the results were plotted as a double-reciprocal (Lineweaver–Burke) plot aromatase substrate [1 β-$^3$H(N)]-androst-4-ene-3,17-dione and the co-factor NAPDH for 1 h resulted in a specific activity of 39.3 ± 1.7 pmoles mg$^{-1}$ h$^{-1}$. Addition of 4-HPR to the reaction caused a dose-dependent decrease in aromatase activity, with a

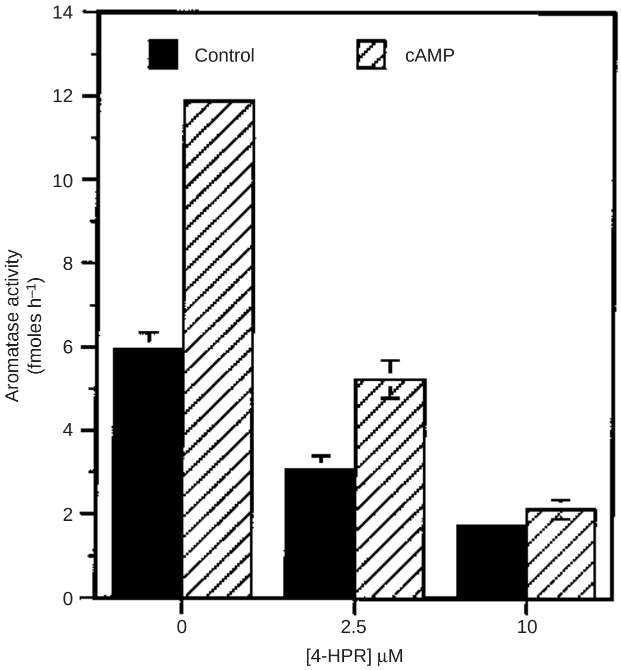

**Figure 3** Effect of 4-HPR on aromatase activity in MCF-7 cells in the absence or presence of cAMP. MCF-7 cells were incubated for 24 h with 25 nM substrate and the indicated concentrations of 4-HPR with or without 1 mM cAMP. Aromatase activity was determined as described. Each bar represents the mean of three determinations ± SE. There was a significant decrease in aromatase activity in cells treated with either concentration of 4-HPR ($P < 0.05$)

1 µM concentration resulting in a 50% decrease in activity ($IC_{50}$) as compared to controls (Figure 1).

The inhibition of microsomal aromatase activity by increasing concentrations of 4-HPR in the presence of different substrate concentrations was also measured and the results were analysed by double-reciprocal (Lineweaver–Burke) plot. As shown in Figure 2, there was a dose-dependent increase in the $K_m$ of the substrate in the presence of 4-HPR. The $V_{max}$ of the enzyme reaction decreased in the presence of 4-HPR.

### Effect of 4-HPR on cellular aromatase activity

When MCF-7 cells were incubated with the aromatase substrate [1β-$^3$H(N)]-androst-4-ene-3,17-dione for 24 h, we observed a specific activity of 5.957 ± 0.399 fmoles h$^{-1}$ million cells$^{-1}$. Co-incubation of the cells with 4-HPR resulted in a dose-dependent decrease in aromatase activity (Figure 3). Treatment of the cells with cAMP, a known inducer of aromatase expression, resulted in an approximately 2-fold increase in specific activity. This was also inhibited in the presence of 4-HPR in a dose-dependent manner (Figure 3).

### Effect of 4-HPR on aromatase mRNA

The amount of aromatase mRNA in MCF-7 cells was measured by semi-quantitative RT-PCR. As seen in Figure 4, treatment of MCF-7 cells with 4-HPR for 24 h had no effect on the basal level of aromatase. Treatment of cells with cAMP for 24 h resulted in an approximately 2-fold increase in aromatase mRNA. This increase was completely abolished in cells co-incubated with 4-HPR.

*© 2000 Cancer Research Campaign*

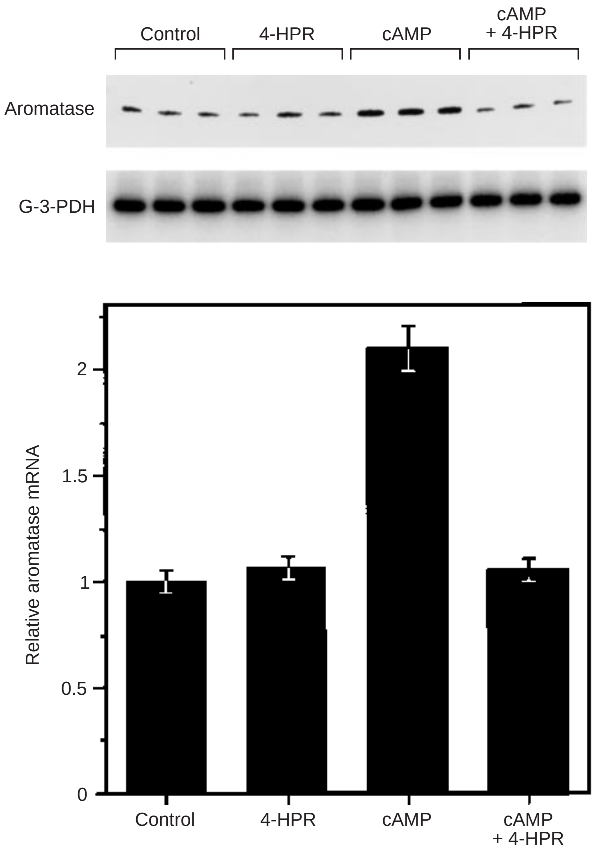

**Figure 4** Effect of 4-HPR on the induction of aromatase mRNA in MCF-7 cells treated with cAMP. MCF-7 cells were incubated with or without (control) 1 mM cAMP in the absence or presence of 10 μM 4-HPR for 24 h. The amount of aromatase mRNA was determined by RT-PCR. For the graph, the level of aromatase mRNA was normalized to the amount of G-3-PDH mRNA, $n = 3 \pm$ SE. The amount of aromatase mRNA was significantly lower in cells co-treated with cAMP and 4-HPR when compared to cAMP alone ($P < 0.05$)

### DISCUSSION

Despite the established chemopreventive effect of 4-HPR towards mammary tumorigenesis, and the pivotal role of aromatase in the growth of breast tumours, the effect of 4-HPR on the activity of aromatase has not, to our knowledge, been studied. As a source of aromatase activity, we isolated microsomes from JEG-3 human choriocarcinoma cells and examined the effect of 4-HPR on aromatase activity. As shown in Figure 1, 4-HPR inhibits microsomal aromatase activity in a dose-dependent manner, with an $IC_{50}$ of 1 μM. The mechanism by which 4-HPR inhibits aromatase activity appears to be multifactorial. Using Lineweaver–Burke analysis of microsomal aromatase activity to determine the kinetics of inhibition, we found that 4-HPR causes an increase in the $K_m$ of the substrate, indicating that 4-HPR competes with the substrate at the substrate binding site (Figure 2). However, the $V_{max}$ of the reaction is decreased in the presence of 4-HPR, indicating that non-competitive inhibition is also at work. Thus, the inhibition of aromatase activity by 4-HPR is complex.

Because aromatase is overexpressed in breast cancer tissue, the effect of aromatase inhibitors in breast cancer cells is of particular interest. We therefore examined the effects of 4-HPR on aromatase activity in MCF-7 human breast cancer cells, an oestrogen-receptor-positive cell line that has been extensively used in aromatase studies (Yano et al, 1995; Zhou et al, 1996). Although these cells did not possess sufficient aromatase to easily detect activity in microsomal preparations, activity in intact cells is measurable with a longer incubation time. As shown in Figure 3, 4-HPR caused a dose-dependent inhibition of both basal and cAMP-induced aromatase activity in MCF-7 cells. Although not as potent an inhibitor of cellular aromatase as some of the recently synthesized substrate analogs of aromatase, which inhibit aromatase activity at nanomolar concentrations (Brueggemeier and Katlic, 1990; Miller, 1996; Yue and Brodie, 1997), 4-HPR has been shown to accumulate preferentially in the breast, where concentrations reach the micromolar range (Formelli et al, 1989). Thus, the inhibition of aromatase activity demonstrated here occurs at pharmacologically relevant doses.

cAMP activates transcription of *CYP19*, which encodes aromatase, and contains cAMP-responsive elements (Zhao et al, 1996; Zhou et al, 1996). Although the results of the microsomal assay (Figures 1 and 2) indicate that direct enzyme inhibition can account for the decrease in aromatase activity in MCF-7 cells, the inhibition of cAMP-induced cellular aromatase activity by 4-HPR demonstrated in Figure 3 may also be the result of modulation of aromatase expression. To test this possibility, we measured the level of aromatase mRNA expression by RT-PCR. As shown in Figure 4, aromatase mRNA is expressed in untreated MCF-7 cells (control). Incubation with 4-HPR did not alter aromatase mRNA, indicating that the inhibition of aromatase activity by 4-HPR in untreated cells shown in Figure 3 is likely the result of direct inhibition of the enzyme. Treatment of the cells with cAMP caused an increase in the expression of aromatase mRNA which was completely blocked in the presence of 4-HPR. The inhibition of aromatase activity in intact MCF-7 cells treated with cAMP therefore results from both direct inhibition and an inhibition of aromatase expression. Since cAMP increases aromatase expression by activating aromatase transcription, this result suggests that 4-HPR may interfere with aromatase transcription, but further experiments are necessary to definitively establish this.

These results suggest that part of the established chemopreventive activity of 4-HPR towards mammary tumourigenesis may be the result of its effects on aromatase activity and expression. The effects of natural retinoids on aromatase are currently under study.

### ACKNOWLEDGEMENTS

The authors wish to thank Dr Grace Chao Yeh for her support and comments on the manuscript, and Dr Angela Brodie for her help.

### REFERENCES

Bahn RS, Worsham A, Speeg KV Jr, Ascoli M and Rabin D (1981) Characterization of steroid production in cultured human choriocarcinoma cells. *J Clin Endocrinol Metab* **52**: 447–450

Bednarek A, Shilkaitis A, Green A, Lubet R, Kelloff G, Christov K and Aldaz CM (1999) Suppression of cell proliferation and telomerase activity in 4-(hydroxyphenyl)retinamide-treated mammary tumours. *Carcinogenesis* **20**: 879–883

Blankenstein MA, van de Ven J, Maitimu-Smeele I, Donker GH, de Jong PC, Daroszewski J, Szymczak J, Milewicz A and Thijssen JH (1999) Intratumoural levels of oestrogens in breast cancer. *Steroid Biochem Mol Biol* **69**: 293–297

Bradford MM (1976) A rapid and sensitive method for the quantitation of microgram quantities of protein utilizing the principle of protein-dye binding. *Anal Biochem* **72**: 248–254

Brodie A, Lu Q and Nakamura J (1997) Aromatase in the normal breast and breast cancer. *J Steroid Biochem Mol Biol* **61**: 281–286

 © 2000 Cancer Research Campaign

Brodie A, Lu Q and Long B (1999) Aromatase and its inhibitors. *J Steroid Biochem Mol Biol* **69**: 205–210

Brueggemeier RW and Katlic NE (1990) Aromatase inhibition by an enzyme-activated irreversible inhibitor in human carcinoma cell cultures. *Cancer Res* **50**: 3652–3656

Choate JV and Resko JA (1996) Paradoxical effect of an aromatase inhibitor, CGS-20267, on aromatase activity in guinea pig brain. *J Steroid Biochem Mol Biol* **58**: 411–415

Costa A, Formelli F, Chiesa F, Decensi A, De Palo G and Veronesi U (1994) Prospects of chemoprevention of human cancers with the synthetic retinoid fenretinide. *Cancer Res* **54**: 2032s–2037s

Crist KA, Wang Y, Lubet RA, Steele VE, Kelloff GJ and You M (1997) Effect of early vs. late administration of 4-hydroxyphenylretinamide (4-HPR) on N-methyl-N-nitrosourea (MNU)-induced mammary tumourigenesis. *J Cell Biochem Suppl* **27**: 92–99

de Cupis A and Favoni RE (1997) Oestrogen/growth factor cross-talk in breast carcinoma: a specific target for novel antioestrogens. *Trends Pharmacol Sci* **18**: 245–251

Favoni RE, de Cupis A, Bruno S, Yee D, Ferrera A, Pirani P, Costa A and Decensi A (1998) Modulation of the insulin-like growth factor-I system by *N*-(4-hydroxyphenyl)-retinamide in human breast cancer cell lines. *Br J Cancer* **77**: 2138–2147

Formelli F, Carsana R, Costa A, Buranelli F, Campa T, Dossena G, Magni A and Pizzichetta M (1989) Plasma retinol level reduction by the synthetic retinoid fenretinide: a one year follow-up study of breast cancer patients. *Cancer Res* **49**: 6149–6152

Formelli F, Clerici M, Campa T, Di Mauro MG, Magni A, Mascotti G, Moglia D, De Palo G, Costa A and Veronesi U (1993) Five-year administration of fenretinide: pharmacokinetics and effects on plasma retinol concentrations. *J Clin Oncol* **11**: 2036–2042

Lippman SM, Heyman RA, Kurie JM, Benner SE and Hong WK (1995) Retinoids and chemoprevention: clinical and basic studies. *J Cell Biochem Suppl* **22**: 1–10

Man T (1994) Solid tumours – chemoprevention with retinoids. *Leukemia* **8**: 1785–1790

Marth C, Bock G and Daxenbichler G (1985) Effect of 4-hydroxyphenylretinamide and retinoic acid on proliferation and cell cycle of cultured human breast cancer cells. *J Natl Cancer Inst* **75**: 871–875

Miller WR and Forrest AP (1976) Oestradiol synthesis from C19 steroids by human breast cancers. *Br J Cancer* **33**: 116–118

Miller WR (1996) Aromatase inhibitors – where are we now? *Br J Cancer* **73**: 415–417

Moon RC and Constantinou A (1997) Dietary retinoids and carotenoids in rodent models of mammary tumorigenesis. *Breast Cancer Res Treat* **46**: 181–189

Prall OW, Rogan EM and Sutherland RL (1998) Oestrogen regulation of cell cycle progression in breast cancer cells. *Steroid Biochem Mol Biol* **65**: 169–174

Sankaranarayanan R and Mathew B (1996) Retinoids as cancer-preventive agents. *IARC Sci Publ* **139**: 47–59

Santner SJ, Pauley RJ, Tait L, Kaseta J and Santen RJ (1997) Aromatase activity and expression in breast cancer and benign breast tissue stromal cells. *Clin Endocrinol Metab* **82**: 200–208

Sasano H and Harada N (1998) Intratumoral aromatase in human breast, endometrial, and ovarian malignancies. *Endocr Rev* **19**: 593–607

Shimodaira K, Fujikawa H, Okura F, Shimizu Y, Saito H and Yanaihara T (1996) Osteoblast cells (MG-63 and HOS) have aromatase and 5 α-reductase activities. *Biochem Mol Biol Int* **39**: 109–116

Simpson ER, Michael MD, Agarwal VR, Hinshelwood MM, Bulun SE and Zhao Y (1997) Cytochromes P450 11: expression of the CYP19 (aromatase) gene: an unusual case of alternative promoter usage. *FASEB J* **11**: 29–36

Wang TT and Phang JM (1996) Effect of N-(4-hydroxyphenyl)retinamide on apoptosis in human breast cancer cells. *Cancer Lett* **107**: 65–71

Yano S, Tanaka M and Nakao K (1995) Anti-tumour effect of aromatase inhibitor, CGS16949A, on human breast cancer cells. *Eur J Pharmacol* **289**: 217–222

Yue W and Brodie AM (1997) Mechanisms of the actions of aromatase inhibitors 4-hydroxyandrostenedione, fadrozole, and aminoglutethimide on aromatase in JEG-3 cell culture. *J Steroid Biochem Mol Biol* **63**: 317–328

Zhao Y, Agarwal VR, Mendelson CR and Simpson ER (1996) Estrogen biosynthesis proximal to a breast tumour is stimulated by PGE2 via cyclic AMP, leading to activation of promoter II of the CYP19 (aromatase) gene. *Endocrinology* **137**: 5739–5742

Zhou D, Clarke P, Wang J and Chen S (1996) Identification of a promoter that controls aromatase expression in human breast cancer and adipose stromal cells. *J Biol Chem* **271**: 15194–15202

Zhou DJ, Pompon D and Chen SA (1990) Stable expression of human aromatase complementary DNA in mammalian cells: a useful system for aromatase inhibitor screening. *Cancer Res* **50**: 6949–6954

*© 2000 Cancer Research Campaign*
